# 20-Hydroxyecdysone Boosts Energy Production and Biosynthetic Processes in Non-Transformed Mouse Cells

**DOI:** 10.3390/antiox13111349

**Published:** 2024-11-02

**Authors:** Oleg Shuvalov, Yulia Kirdeeva, Elizaveta Fefilova, Alexandra Daks, Olga Fedorova, Sergey Parfenyev, Alexander Nazarov, Yulia Vlasova, George S. Krasnov, Nick A. Barlev

**Affiliations:** 1 Institute of Cytology of the Russian Academy of Sciences, St. Petersburg 194064, Russia; yulia.kirdeeva@yandex.ru (Y.K.); e.fefilova@list.ru (E.F.); alexandra.daks@gmail.com (A.D.); fedorovaolga0402@gmail.com (O.F.); gen21eration@gmail.com (S.P.); anazarov2021@gmail.com (A.N.); 2 Almazov National Medical Research Centre, St. Petersburg 197341, Russia; vlasovayu@rambler.ru; 3 Engelhardt Institute of Molecular Biology, Russian Academy of Sciences, Moscow 119991, Russia; gskrasnov@mail.ru; 4 Department of Biomedical Sciences, School of Medicine, Nazarbayev University, Astana 010000, Kazakhstan

**Keywords:** 20-hydroxyecdysone (ecdysterone), glycolysis, oxidative phosphorylation, one-carbon metabolism, ATP production, C2C12, c-Myc, ATF3, reactive oxygen species

## Abstract

20-Hydroxyecdysone (20E) is an arthropod steroid hormone that possesses a number of beneficial pharmacological activities in humans, including anabolic, antioxidant, hypoglycemic, cardioprotective, hepatoprotective, neuroprotective, and antineoplastic properties, etc. While several studies have explored the anabolic activity of 20E in muscle cells, they have concentrated on its effects on myofibril size, protein biosynthesis intensity, and myostatin expression, without assessing energy metabolism. In this research, we have demonstrated that 20E boosts both catabolism and anabolism, coupling energy-producing and biosynthetic metabolic processes in mouse myoblasts and fibroblasts in the same way. Using a transcriptomic approach, we identified the 20E-mediated up-regulation of genes involved in different metabolic processes. Further experiments revealed that 20E increased the levels of enzymes involved in glycolysis and one-carbon metabolism. It also increased the uptake of glucose, glycolysis, respiration, the production of ATP, and global protein biosynthesis in mouse myoblasts and fibroblasts. This phenomenon involves the PI3K/AKT/mTOR signaling pathway. Taken together, the observed 20E-dependent upregulation of energy metabolism may be the main reason for 20E’s well-known anabolic activity.

## 1. Introduction

20-hydroxyecdysone (ecdysterone, 20E) is one of the main hormones that controls the moulting and metamorphosis of arthropods. It is also synthesized by a number of plant species, apparently for protection against feeding by insects [1,2].

Importantly, 20E displays multiple beneficial pharmacological activities in humans, including anabolic, antioxidant, hypoglycemic, cardioprotective, hepatoprotective, neuroprotective, and other properties [3,4,5,6]. According to its pharmacological classification, 20E can be referred to as an adaptogenic substance that can increase “nonspecific” resistance to adverse environmental influences on the body, as well as the body’s resistance to stress, similar to ginsenosides from Panax sp. or biologically active compounds derived from Eleutherococcus senticosus, Schisandra chinensis, and Rhodiola rosea [7,8,9,10,11].

20E has been studied in a number of clinical trials to treat numerous disorders (summarized in [3]). Additionally, 20E has been utilized in multiple clinical trials by the French company “BioPhytis” to treat Dushen’s myodystrophy, Alzheimer’s disease, sarcopenia, and the severe distress syndrome that developed following the COVID-19 outbreak (https://www.biophytis.com/ (accessed on 22 March 2024)) [12]. According to the results of a randomized, placebo-controlled, phase 2/3 trial, 20E significantly reduced the risk of death or respiratory failure in adults hospitalized with severe respiratory symptoms due to COVID-19 [13].

20E is regarded as a compound that is non-toxic to mammals, with an LD50 in mice of approximately 9 g/kg of weight [3,14]. Moreover, Leuzea cartamoides, the traditional medicinal plant of the Altai area of Russia, contains a significant amount of 20E and has been utilized there for centuries as an adaptogenic substance [15]. Furthermore, clinical trials with 20E have demonstrated that this compound has a safe profile [3].

Worldwide, different manufacturers sell 20E as dietary supplements in the form of plant extracts of varying purity. However, the majority of 20E-containing supplements are derived from the Chinese plant Cyanotis sp., the safety of which remains in question [16].

A number of in vitro and in vivo studies have demonstrated the 20E-mediated improvement of lipid and glucose metabolism in the liver and the heart [17,18,19,20,21]. Moreover, 20E is best known for its mild, natural anabolic activity that does not affect androgen receptors [3,4]. Regarding muscle cells, the studies were limited in monitoring the effects of 20E on the size of myotubes, intensity of protein biosynthesis, and myostatin expression [4,22,23,24]. Notably, none of these reports investigated the effects of 20E on energy metabolism in muscle cells with respect to the analysis of glycolysis and respiration.

In the present study, we have combined transcriptomic analysis with a SeaHorse energy profiling approach to study the impact of 20E on the energy metabolism of 20E-responsive mouse C2C12 myoblasts. In parallel, another mouse mesodermal non-transformed cell line, NIH/3T3 fibroblasts, was used. We uncovered a number of 20E-responsive genes involved in metabolism and their respective transcriptional regulators. Furthermore, the functional consequences of the 20E-mediated effect on these cells included the up-regulation of glycolysis and respiration, enhanced glucose uptake, and ATP production. Moreover, the observed phenomena included the 20E-dependent increase in global protein biosynthesis, which was depended on the PI3K/AKT/mTOR signaling pathway. These results correspond well with the literature data.

## 2. Materials and Methods

### 2.1. Cell Lines and Reagents

Mouse myoblasts C2C12 and embryonic fibroblasts NIH/3T3 were purchased from ATCC. They were cultured in DMEM medium, supplemented with 10% FBS, 100 μg/mL gentamycin, and 2 mM L-glutamine at 37 °C in a 5% CO_2_ atmosphere.

20-hydroxyecdysone (20E, β-ecdysterone) (European Pharmacopoeia (EP) Reference Standard, Sigma, St. Louis, MO, USA, 98% purity) was dissolved in DMSO. All experiments with 20E used DMSO as a control.

### 2.2. RNA-Seq Analysis

For RNA-seq analysis, C2C12 myoblasts were treated with 1 μM of 20E for 24 h. A DMSO treatment was applied to the control cells. For every sample, six biological replicates were employed. Using a MagNA Pure Compact instrument (Roche, Basel, Switzerland), total RNA was extracted using the MagNA Pure Compact RNA Isolation Kit (Roche, Switzerland). A Qubit 4 fluorometer (Thermo Fisher Scientific, Waltham, MA, USA) was used to determine the concentration of RNA, and an Agilent 2100 Bioanalyzer (Agilent Technologies, Santa Clara, CA, USA) was used to evaluate the quality of the RNA. The extracted RNA had an RNA integrity number (RIN) of at least 9.5.

Following the manufacturer’s recommendations, 1 μg of total RNA was used to construct cDNA libraries using the TruSeq Stranded mRNA Library Prep Kit (Illumina, San Diego, CA, USA). On an Illumina NextSeq 2000 System, libraries were sequenced in single-read mode (51 bp). For every sample, at least 20 million readings were produced.

Trimmed reads were mapped to the mouse genome GRCm39 (Ensembl release 110) with STAR 2.7.2b [25]. The 5′-to-3′ transcript coverage profiles (3′-bias) were analyzed with the geneBody_coverage script from the RSeQC 3.0.1 toolkit [26]. Read counts per gene were calculated with featureCounts [27], RSEM 1.3.0 [28], and Salmon [29]. Differential expression and downstream analyses were performed with the edgeR 3.36.0 [30], clusterProfiler 4.2.2, topGO 2.46.0, and pathview 1.34 R packages [31], the STRINGdb database [32], and proprietary tools.

We observed a strong correlation (Spearman r = −0.42) between the log2 transcript length (LogTL) and the log2 expression fold change (LogFC) when comparing the E20-treated samples to the controls. This situation usually occurs in the case of RNA degradation and the consequent differences in 3′-bias profiles between the comparison groups. However, (1) 3′-bias differences were completely absent between any samples; and (2) the RIN values for all samples were 9 or higher. We recalculated the data using RSEM and Salmon with all enabled bias corrections, as well as by 3′-tail transcript regions (500 bp) as described in [33], but this did not eliminate the dependence between the LogFC and LogTL. Thus, the reasons for this dependence remain unclear and may be associated with a natural biological effect derived from exposure to 20E. However, being unsure of this, we decided to proceed cautiously, as follows: we eliminated the dependence between LogFC and LogTL by subtracting the approximate straight line from the linear regression. Then, we took the minimum absolute value of the initial LogFC and the corrected LogFC. We considered the LogFC to be zero if their signs differed.

We considered differentially expressed genes with FDR ≤ 0.05 and LogFC ≥ 0.1.

### 2.3. Relative Real-Time PCR

Total RNA was extracted using a TRIzol-based reagent (Evrogen, Moscow, Russia) following the manufacturer’s instructions. The RevertAid First-Strand cDNA Synthesis Kit (Evrogen, Moscow, Russia) was utilized to initiate reverse transcription with oligo d(T) primer on three micrograms of total RNA. Using a CFX 1000 PCR machine (BioRad, Hercules, CA, USA) and SYBR green mix (Evrogen, Moscow, Russia), real-time PCR was performed in triplicate. The software CFX Manager (Version 3.1) was used to analyze the results. The reference gene selected was β-actin. The 2^−∆∆Ct^ technique was used to calculate the relative expression. Table S1 provides a list of primer sequences.

### 2.4. Western Blot

Cells were lysed in RIPA buffer (150 mM NaCl; 50 mM Tris-HCl pH 7.5; 0.3% Triton-X100; 1 mM PMSF, with protease inhibitor cocktail) and then sonicated in preparation for immunoblotting. A BCA assay (ThermoScientific, Waltham, MA, USA) was used to measure the protein content. To balance the concentration, samples were then diluted using a Laemli buffer. After 25 μg of protein lysate samples were run in 13% SDS-PAGE (TRIS-Glycine running buffer), they were transferred to a PVDF membrane (Bio-RAD, Hercules, CA, USA), which was then blocked with 5% nonfat milk in PBST and incubated with primary antibodies overnight, which included the following: c-Myc (D84C12, Cell Signaling, Danvers, MA, USA), HK2 (MA5-14849, ThermoScientific, Waltham, MA, USA), LDHA (GB11342, ServiceBio, Wuhan, China), SHMT2 (DF6347, Cloud-Clone, Wuhan, China), MTHFD2 (DF12213, Cloud-Clone, Wuhan, China), p-mTOR (#2971; Cell Signaling, Danvers, MA, USA), mTOR (ab25580; Abcam, Waltham, MA, USA), p-AKT (#2971; Cell Signaling, Danvers, MA, USA), AKT (#9272; Cell Signaling, Danvers, MA, USA), AldoA (sc-377058, Santa-Cruze, Santa-Cruze, CA, USA), anti-puromycin (MABE 343, 1:1000, Millipore, Burlington, MA, USA), and β-actin (#8457, Cell Signaling, Danvers, MA, USA). Following multiple PBST washes, we applied secondary anti-mouse or anti-rabbit antibodies (1:10,000; Sigma-Aldrich, St. Louis, MO, USA) conjugated with horseradish peroxidase. The results were detected using the ECL system (ThermoScientific, Waltham, MA, USA) and the ChemiDoc Touch Imager (Bio-Rad, Hercules, CA, USA).

Three biological repeats were carried out for each experiment. The software Image J (Version 1.51) was used to perform the quantification. The representative images of Western blots that were repeated three times with essentially the same results (see the graphs in the figures) are demonstrated. The protein/actin ratio was calculated and is listed in the graphs. Results are presented as the means ± SEM of the three biological experiments. Asterisks indicate a significant difference in protein expression (* *p* ≤ 0.05; ** *p* ≤ 0.01; n.s.—non-significant).

### 2.5. SeaHorse Energy Profiling

For the accurate quantification of glycolysis and respiration, the Seahorse XFe24 machine (Agilent, Santa Clara, CA, USA) and the Seahorse XF Real-Time ATP Rate Assay Kit (Agilent, Santa Clara, CA, USA) were used in accordance with the manufacturer’s instructions. For these experiments, 30,000 cells per well were seeded on 24-well SeaHorse plates in five replicates and treated with 1–100 μM of 20E for 24 h. The final concentrations of both oligomycin and the Rotenone/Antimycin A mix were 3 µM. To quantify glyco- and mitoATP production, the SeaHorse XF Real-Time ATP Rate Assay Excel plugin (Agilent, Santa Clara, CA, USA) was used. Results are presented as the mean ± SEM.

### 2.6. ATP Production Assay

To directly quantify the level of ATP, 100,000 cells per well were seeded on a 12-well plate and treated with different concentrations of 20E for 24 h, followed by using the ATP Assay Colorimetric Kit (Abcam, ab83355, Waltham, MA, USA) in accordance with the manufacturer’s instructions. The experiment was carried out in triplicate. Results are presented as the percentage of ATP level relative to the control (DMSO-treated cells) and given as the mean ± SEM.

### 2.7. Glucose Consumption Assay

A total of 20,000 cells were seeded in each well of a 96-well plate, and the cells were exposed to varying doses of 20E for 24 h. The Glucose Uptake Assay Kit (Abcam, ab136955, Waltham, Boston, MA, USA) was used in accordance with the manufacturer’s instructions on the day of analysis. The experiment was carried out in triplicate. The results are shown as the mean ± SEM and as a percentage of glucose uptake intensity in comparison to the control group (cells treated with DMSO).

### 2.8. Surface Sensing of Translation (SUnSET) Assay

A SUnSET assay was applied to assess the intensity of global protein biosynthesis [34]. A total of 30,000 cells were seeded in 3 cm cell culture dishes one day before analysis. First, cells were treated for 15 min with 10 μg/mL puromycin. In this stage, puromycin—an analogue of tyrosyl-trna—was integrated into developing polypeptide chains to show the translation level in real time within the cells. Then, the cells were collected and subjected to immunoblotting with anti-puromycin antibodies.

### 2.9. Statistical Analysis

We used Student’s *t*-test to search for pairwise statistically significant differences in experiments with N = 3. For the SeaHorse energy profiling experiments with N = 5, the Mann–Whitney (*U*-test) was applied. GraphPad Prism 8 was used to perform the analysis. A statistical difference was defined as *p* < 0.05. * *p* < 0.05; ** *p* < 0.01; *** *p* < 0.001; non-significant, n.s.

## 3. Results

### 3.1. Transcriptomic Analysis of 20E-Treated C2C12 Myoblasts

First of all, to elucidate the impact of 20E on gene expression, we carried out the RNA-seq assay. To do this, we treated the C2C12 myoblast cell line with 1 µm of 20E for 24 h.

**Figure 1:**
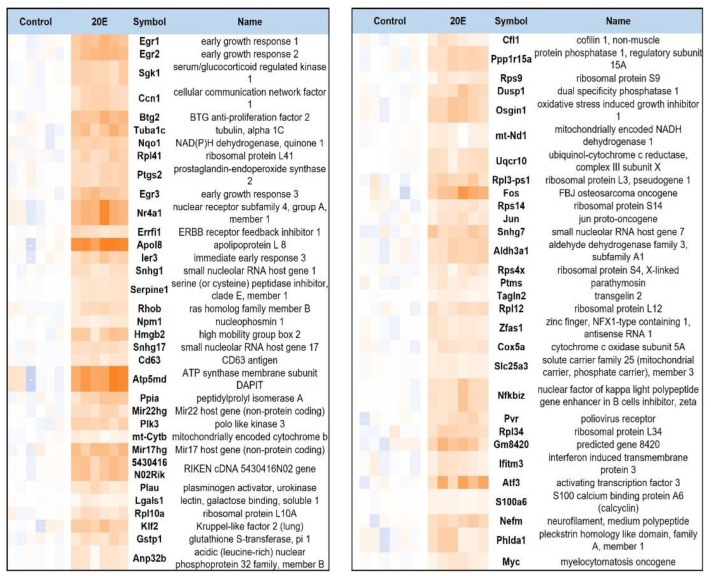
Top 70 genes up-regulated by treatment of C2C12 myoblasts with 1 μM of 20E for 24 h (RNA-seq data). Control samples treated with DMSO only; 20E samples treated with 20E.

Interestingly, based on the LogFC values (Table S2), it seems that 20E preferentially increased rather than repressed the expression of genes. In total, we revealed 768 differentially expressed genes, 424 of which were up-regulated and 344 which were down-regulated (Table S2). Interestingly, the list of the top 70 up-regulated genes (Figure 1) includes AP-1 (activating protein-1) transcription factor components: Jun (transcription factor Jun), Fos (transcription factor Fos), Atf3 (Activating Transcription Factor 3); its downstream effectors: transcription factors Egr1 (early growth response 1), Egr2 (early growth response 2), and Egr3 (early growth response 3); components of the mitochondrial respiration chain: mt-Nd1 (NADH dehydrogenase I) and Nqo1 (NAD(P)H dehydrogenase, quinone 1)–complex I; cytochrome b; Uqcr10 (Ubiquinol-cytochrome c reductase, subunit X) and Cox5a (Cytochrome c oxidase subunit 5A)–complex III; and Atp5md (ATP synthase membrane subunit DAPIT)–complex V. Moreover, two transcriptional factors that are critical for global metabolic regulation—c-Myc and ATF3—were identified among the top genes up-regulated by 20E (Figure 1).

**Figure 2:**
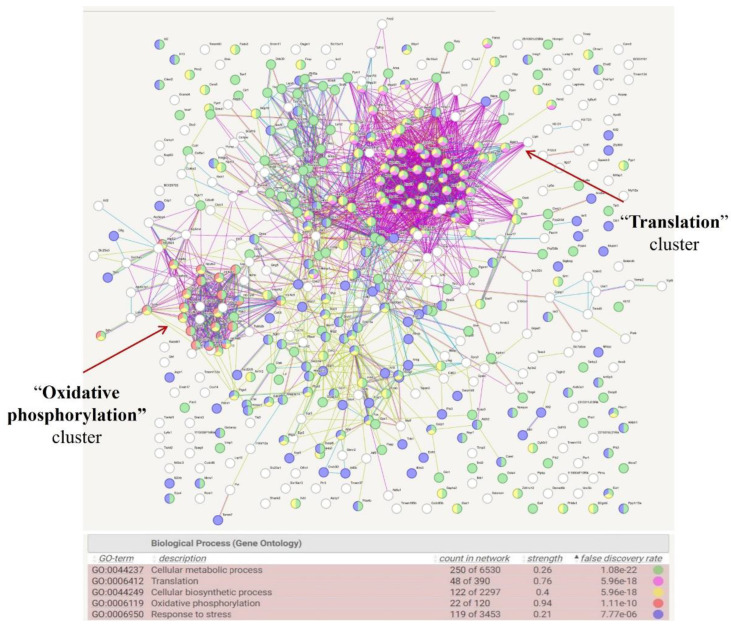
The analysis of the protein–protein interaction network for up-regulated genes using the STRING database (https://string-db.org/; accessed on 13 February 2024). Colors indicate protein participation in the corresponding cellular process. Two large clusters of proteins stand out: “oxidative phosphorylation” and “translation”.

STRING-based analysis of the up-regulated genes revealed the significantly enriched protein–protein interaction (PPI) network (PPI enrichment *p*-value: < 1.0 × 10^−16^), which includes genes involved in cellular metabolic processes (large sets of genes involved in oxidative phosphorylation and cellular biosynthetic processes), translation, and response to stress (Figure 2). The KEGG PATHWAY database profiling analysis of up-regulated genes (https://www.genome.jp/kegg/pathway.html; accessed on 12 February 2024) also showed that about 30 genes code for parts of all mitochondrial respiratory complexes (Figure S1). These data fit with the well-known pharmacological properties of 20E, including its anabolic and adaptogenic properties [3].

In turn, the analysis of down-regulated genes (Figure S2) revealed weak PPI enrichment (*p*-value: 0.107). However, this list includes genes involved in the negative regulation of cellular metabolic processes and genes that are expressed in embryonic tissues (Figure S3).

Taken together, these data point to a global impact of 20E on metabolic networks in C2C12 myoblasts, including the up-regulation of biosynthetic processes, translation, and oxidative phosphorylation.

### 3.2. 20E Up-Regulates Enzymes of Glycolysis and One-Carbon Metabolism

As we observed the 20E-mediated transcriptional up-regulation of different metabolic genes, including c-Myc and ATF3, which are the two master regulators of the metabolic network, we chose to focus on the effects of 20E on glycolysis and one-carbon metabolism. These metabolic pathways are important biochemical “hubs” that sustain a variety of biosynthetic processes and are directly regulated by c-Myc and ATF3 [35,36,37,38,39,40].

For this purpose, we used both the mouse C2C12 myoblasts and the mouse NIH/3T3 embryonic fibroblast cell line to see how 20E changed the expression of important enzymes for glycolysis and one-carbon metabolism.

To this end, we treated C2C12 and NIH/3T3 cells with an extended concentration range of 20E (0.1–100 µM) for 24 h, followed by real-time PCR and Western blotting. We looked at the expression levels of glut1, hk2, and ldha genes, which code for glucose transporter 1 (Glut1), hexokinase 2 (HK2), and lactate dehydrogenase A (LDHA), respectively. To assess the effect of 20E on one-carbon metabolism, we measured the protein expression of psat1, phgdh, and psph, as well as shmt2 and mthfd2, encoding three enzymes of the serine biosynthesis pathway (Phosphoserine Aminotransferase 1, Phosphoglycerate Dehydrogenase, and Phosphoserine Phosphatase) and two key mitochondrial enzymes of the folate cycle (Serine hydroxymethyltransferase 2 and Methylenetetrahydrofolate Dehydrogenase 2). Beyond metabolic enzymes, we also studied the expression of c-Myc and ATF3 as two important transcriptional regulators of glycolysis, one-carbon metabolism, and respiration.

**Figure 3:**
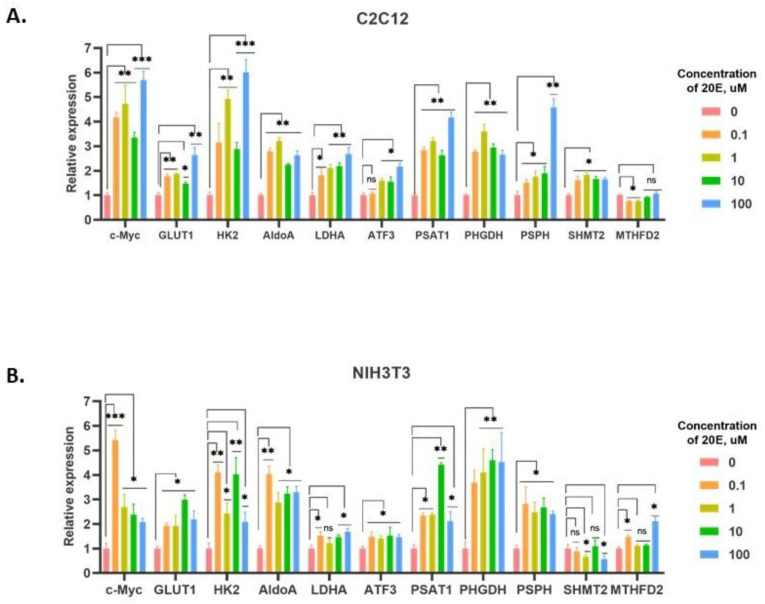
20E enhances the expression of genes that code for glycolysis enzymes (GLUT1—Glucose Transporter 1; HK2—Hexokinase 2; LDHA—Lactate Dehydrogenase (**A**), one-carbon metabolism enzymes (PSAT1—Phosphoserine Aminotransferase 1; PHGDH—Phosphoglycerate Dehydrogenase; PSPH—Phosphoserine phosphatase; SHMT2—Serine Hydroxymethyltransferase 2; MTHFD2—Methylenetetrahydrofolate Dehydrogenase 2), and their respective transcriptional regulators (c-Myc—v-Myc avian myelocytomatosis viral oncogene homolog; ATF3—Activating transcription factor 3). (**A**). C2C12 cells. (**B**). NIH3T3 cells. Real-time PCR data shows the expression of the target genes and was calculated relative to the control (DMSO-treated) cells. β-actin was chosen as the reference gene. Student’s *t*-test was applied to check pairwise statistically significant differences. * *p* ≤ 0.05; ** *p* ≤ 0.01; *** *p* ≤ 0.001.

The relative real-time PCR data showed that 20E significantly up-regulated the expression of all the genes studied in both cell lines (Figure 3). Importantly, 20E-induced expression of metabolic genes was not concentration-dependent, and the effect was observed in the whole range of concentrations (0.1–100 µM) tested.

**Figure 4:**
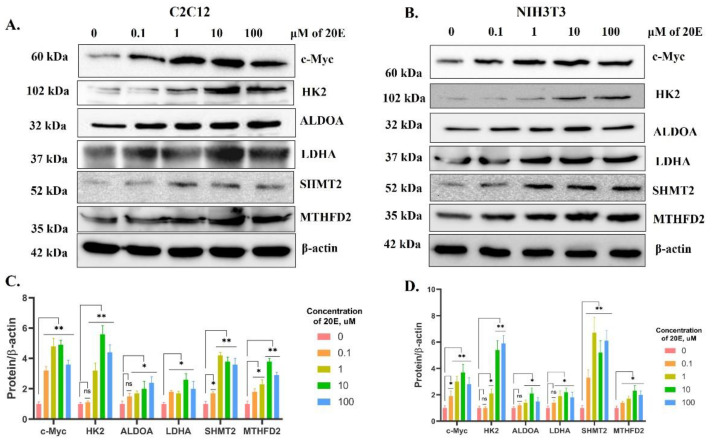
20E up-regulates enzymes involved in glycolysis, one-carbon metabolism, and their respective transcriptional regulators at the protein level (Western blot). (**A**,**C**). C2C12 cells. (**B**,**D**). NIH3T3 cells. The values shown are the means ± SEM of the three biological experiments. The software Image J was used to perform the quantification. Student’s *t*-test was applied to check pairwise statistically significant differences. Asterisks indicate a significant difference in protein expression (* *p* ≤ 0.05; ** *p* ≤ 0.01), ns—non significant.

Importantly, the 20E-mediated augmentation of the studied enzymes and their transcriptional regulators was also confirmed at the protein level. As seen in Figure 4, 20E significantly increased HK2, LDHA, SHMT2, MTHFD2, and c-Myc in both cell lines.

These data demonstrate the positive impact of 20E on glycolysis and one-carbon metabolism in both C2C12 and NIH3T3 cells.

### 3.3. 20E Increased Glycolysis and Respiration

We looked at how 20E affected glucose uptake and ATP production in C2C12 myoblasts and NIH/3T3 fibroblasts to learn more about how it affected energy metabolism. We quantified the glucose uptake and ATP production rate in cells treated with 0.1–100 µm of 20E for 24 h using the glucose uptake assay (Abcam) and ATP assay (Abcam) kits, respectively.

**Figure 5:**
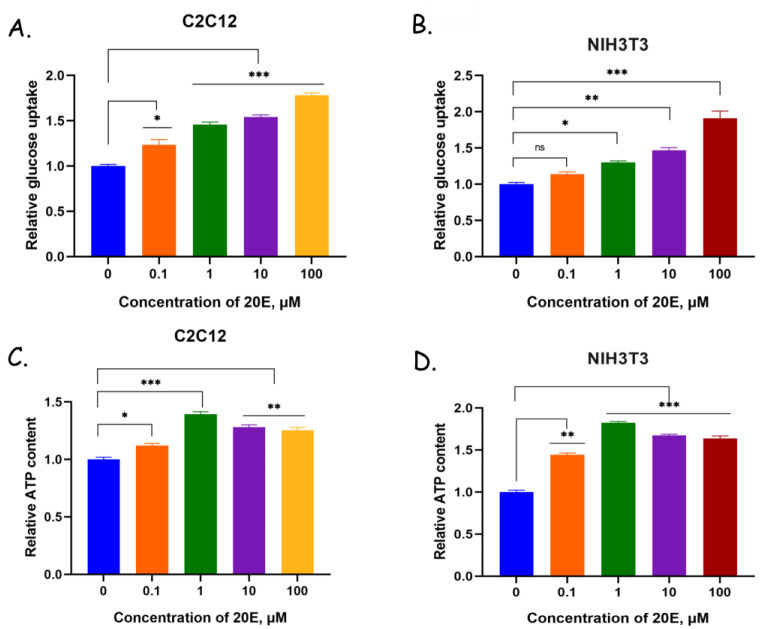
20E increased glucose uptake in (**A**) C2C12 and (**B**) NIH/3T3 cells, and ATP content in (**C**) C2C12 and (**D**) NIH/3T3 cells. The glucose uptake kit (Abcam) and ATP assay kit (Abcam) were used. Student’s *t*-test was applied to check pairwise statistically significant differences. * *p* ≤ 0.05; ** *p* ≤ 0.01; *** *p* ≤ 0.001.

The results in Figure 5A,B show that 20E increased glucose uptake by up to 1.5 times. This is consistent with the increased expression of Glut1. In addition, 20E increased ATP production 1.2–1.5 times in both cell lines studied (Figure 5C,D).

**Figure 6:**
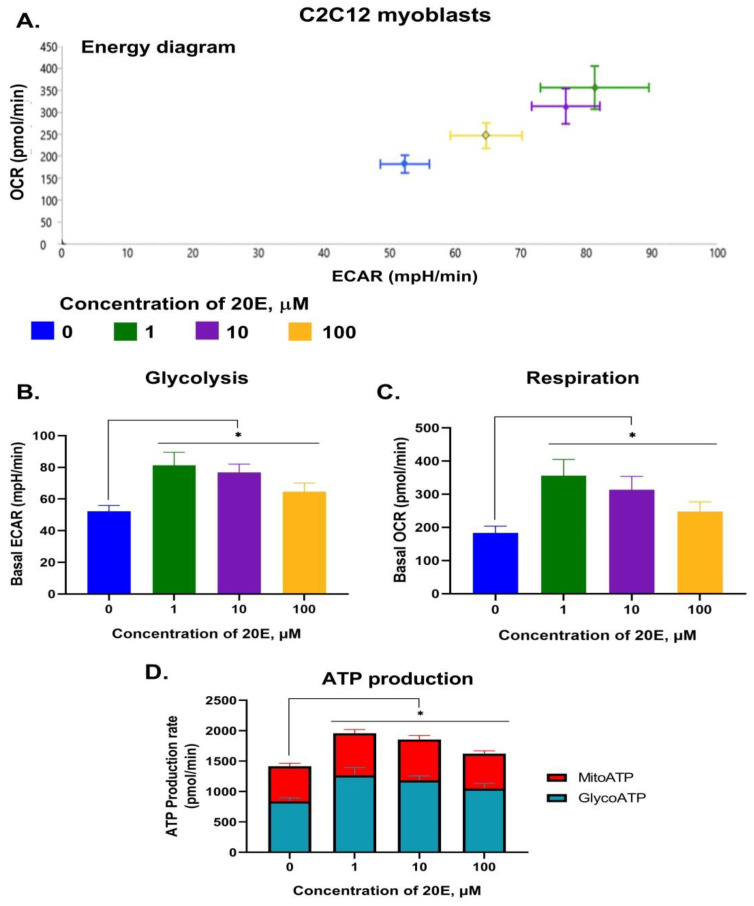
20E augments glycolysis, respiration, and ATP production in C2C12 cells. SeaHorse Data. (**A**). The energy diagram reflects the intensity of glycolysis (ECAR) and respiration (OCR). Diagrams showing the intensity of glycolysis (**B**) and respiration (**C**). (**D**). The rate of Mito- and GlycoATP production. The SeaHorse XF Real-Time ATP Rate Assay Kit was applied. OCR—Oxygen Consumption Rate (shows respiration), ECAR—Extracellular Acidification Rate (shows glycolysis). A Mann–Whitney (*U*-test) was applied to check statistically significant differences. * *p* ≤ 0.05.

**Figure 7:**
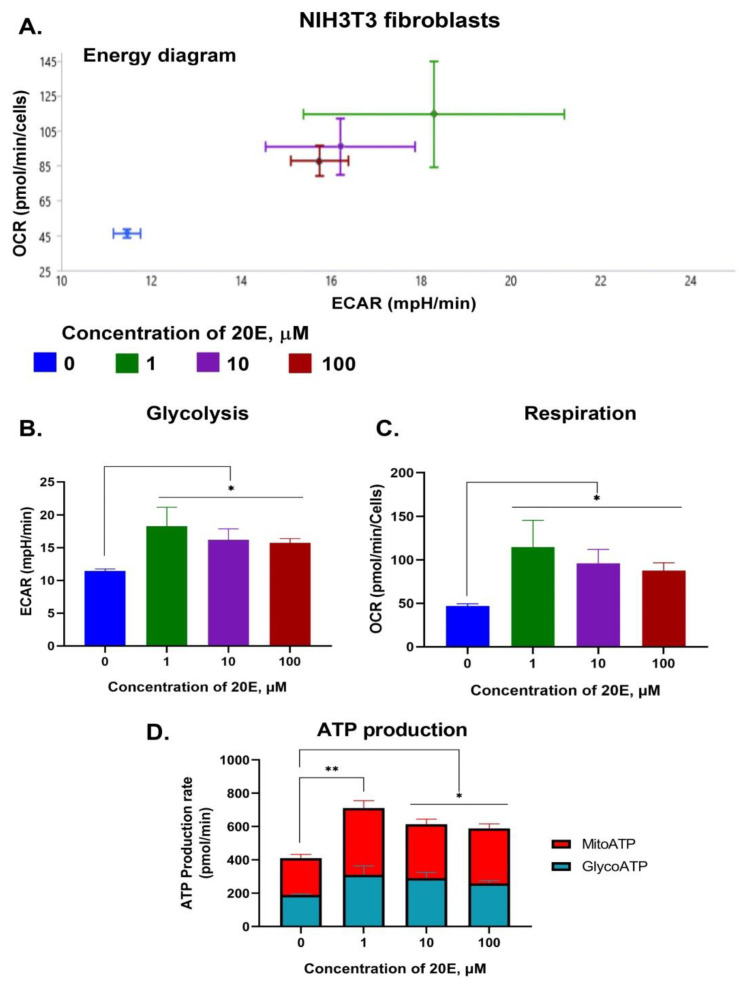
20E augments glycolysis, respiration, and ATP production in NIH3T3 cells. SeaHorse Data. (**A**). The energy diagram reflects the intensity of glycolysis (ECAR) and respiration (OCR). Diagrams showing the intensity of glycolysis (**B**) and respiration (**C**). (**D**) The rate of Mito- and GlycoATP production. The SeaHorse XF Real-Time ATP Rate Assay Kit was employed. OCR—Oxygen Consumption Rate (shows respiration), ECAR—Extracellular Acidification Rate (shows glycolysis). A Mann–Whitney (*U*-test) was applied to check statistically significant differences * *p* ≤ 0.05; ** *p* ≤ 0.01.

To further elucidate the impact of 20E on glycolysis and respiration, we carried out SeaHorse energy profiling using an ATP real-time rate assay kit (Agilent, USA). We observed a significant 20E-mediated induction of glycolysis and, especially, respiration in both cell lines (Figure 6, Figure 7 and Figure S4). The greatest increase in respiration (up to 1.5 and 2 times for C2C12 and NIH/3T3, respectively) was observed upon treatment with 1 μM of 20E, followed by 10 μM and 100 μM. The same tendency was also true for glycolysis, which was up-regulated by 1.6 times. Using SeaHorse to look at ATP production revealed that, even though 20E increased ATP production in both glycolysis and oxidative phosphorylation (glycoATP and mitoATP), it increased glycoATP production more in C2C12 myoblasts and increased mitoATP production more in fibroblasts (Figure 6D and Figure 7D). Despite this small difference, it can be concluded that, in general, 20E affects myoblasts and fibroblasts in the same way, causing an increase in their energy metabolism.

Taken together, these data clearly demonstrate 20E-induced glucose uptake, glycolysis, respiration, and ATP production, which are all fully consistent with the RNA-seq data, real-time PCR, and Western blot results, indicating the up-regulation of energy metabolism.

### 3.4. 20E Increased Global Protein Biosynthesis

In previous studies, several groups of researchers have shown that 20E increases protein synthesis in differentiated C2C12 myotubes [22,24], hence having an anabolic effect. After we observed the 20E-dependent up-regulation of genes involved in translation and cellular biosynthetic processes (Figure 3) and revealed the increase of both catabolic and anabolic processes, we decided to also check the impact of 20E on global protein biosynthesis in C2C12 myoblasts and NIH3T3 fibroblasts.

To address this question, we carried out the SUnSET assay, based on the incorporation of puromycin into newly synthesized, nascent peptides because of its structural similarity to tyrosyl-tRNA [34]. We treated C2C12 and NIH3T3 cells with 0.1–100 µM of 20E for 4 h, followed by a 30 min incubation with puromycin and Western blot with anti-puromycin antibodies.

**Figure 8:**
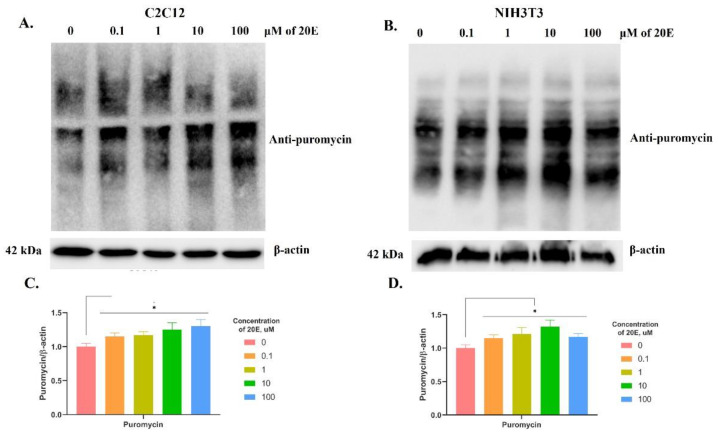
20E increased global protein biosynthesis (Western blot). The results of the SUnSET assay are demonstrated. (**A**,**C**). C2C12 cells. (**B**,**D**). NIH3T3 cells. The values shown are the means ± SEM of three biological experiments. The software Image J was used to perform the quantification. Asterisks indicate a significant difference in protein expression. Student’s *t*-test was applied to check pairwise statistically significant differences. Asterisks indicate a significant difference in protein expression (* *p* ≤ 0.05).

According to the Western blot results (Figure 8), 20E significantly elevates global protein biosynthesis by 15–32%, depending on the concentration and cell line. These results are fully consistent with both the RNA-seq data and the literature data.

### 3.5. 20E-Mediated Up-Regulation of Energy Metabolism Depends on PI3K/AKT Activation

Although 20E possesses well-documented pleiotropic beneficial properties, the cognate molecular mechanisms of 20E-mediated pharmacological activities in mammals remain elusive. However, several research groups found that 20E can activate AKT kinase in a variety of tissues from insects and mammals [17,20,22]. Thus, in the present study, we were tasked with checking if the 20E-mediated up-regulation of energy metabolism depends on PI3K/AKT activity.

**Figure 9:**
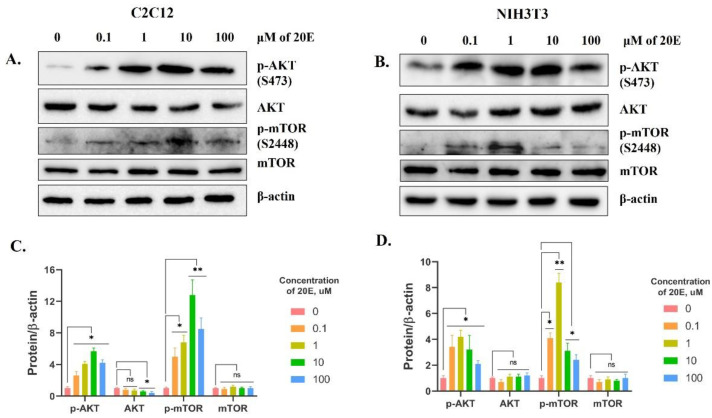
20E activates AKT and mTOR in C2C12 and NIH/3T3 cells. Western blot. (**A**,**C**). C2C12 cells. (**B**,**D**). NIH3T3 cells. The values shown are the means ± SEM of three biological experiments. The software Image J was used to perform the quantification. Student’s *t*-test was applied to check pairwise statistically significant differences. Asterisks indicate a significant difference in protein expression. (* *p* ≤ 0.05; ** *p* ≤ 0.01; ns—non-significant).

To do this, we first treated C2C12 and NIH/3T3 cells with 0.1 to 100 µM of 20E for 24 h. Then, we carried out a Western blot with antibodies that target phospho-AKT and phospho-mTOR. The results shown in Figure 9 demonstrate that 20E induced the accumulation of phospho-AKT (S473) in both cell lines. Importantly, the master regulator of anabolic processes, mTOR, was also activated, because we detected its active form, phospho-mTOR (S2448), which results from phosphorylation by AKT. These data imply the activation of the PI3K/AKT/mTOR pathway.

Then, to confirm the involvement of PI3K/AKT in the 20E-induced up-regulation of energy metabolism, we pre-treated C2C12 and NIH/3T3 cells with the well-known PI3K inhibitor LY294002 (20 µM), which prevents AKT activation, followed by incubation with 1 µM of 20E for 24 h and SeaHorse energy profiling with the SeaHorse ATP real-time production assay kit.

**Figure 10:**
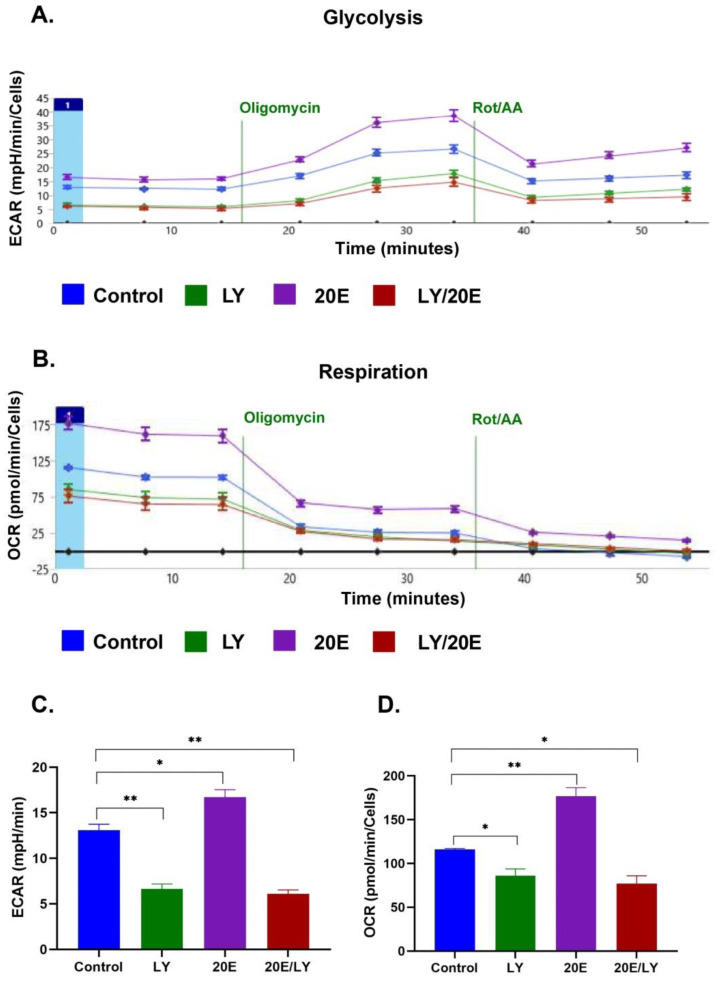
The 20E-mediated increase in glycolysis and respiration in C2C12 cells depends on the PI3K/AKT signaling pathway. SeaHorse data. (**A**). SeaHorse plot showing the intensity of glycolysis. (**B**)**.** SeaHorse plot showing the intensity of respiration. (**C**). Diagram showing the intensity of glycolysis (**D**). Diagram showing the intensity of respiration. SeaHorse XF Real-Time ATP Rate Assay Kit was applied. OCR—Oxygen Consumption Rate (shows respiration), ECAR—Extracellular Acidification Rate (shows glycolysis). Oligomycin—the inhibitor of ATP synthase (respiratory chain Complex V); Rot/AA—the mixture of Rotenone (a respiratory chain Complex I inhibitor) and Antimycin A (a respiratory chain Complex III inhibitor). Mann–Whitney (*U*-test) was applied to check statistically significant differences * *p* ≤ 0.05; ** *p* ≤ 0.01.

**Figure 11:**
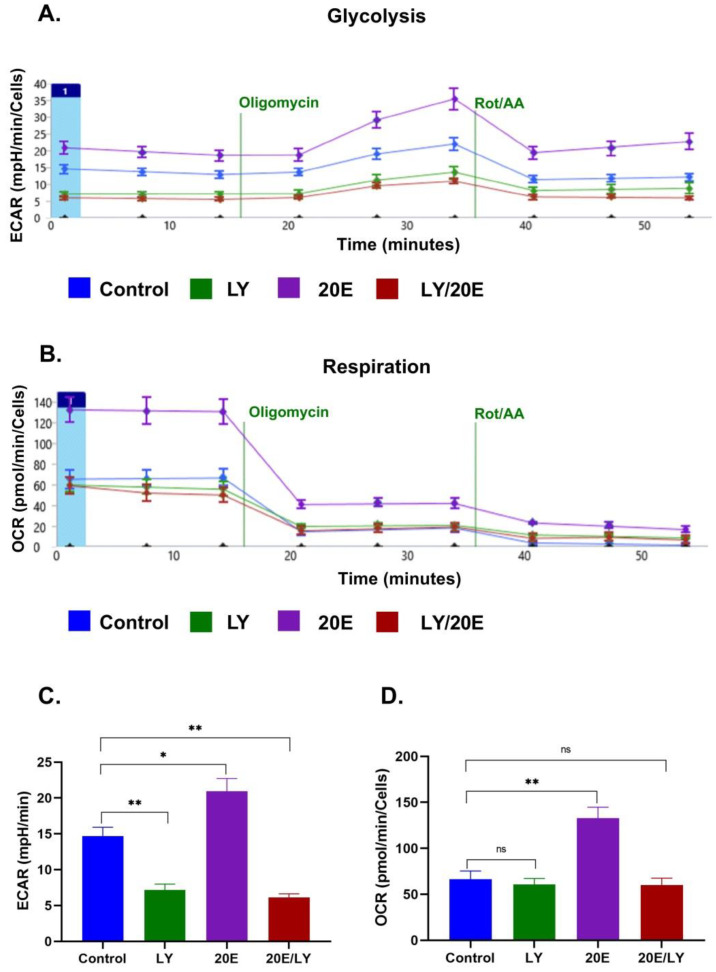
The 20E-mediated increase in glycolysis and respiration in NIH3T3 cells depends on the PI3K/AKT signaling pathway. SeaHorse data. (**A**). SeaHorse plot showing the intensity of glycolysis. (**B**)**.** SeaHorse plot showing the intensity of respiration. (**C**). Diagram showing the intensity of glycolysis (**D**). Diagram showing the intensity of respiration SeaHorse XF Real-Time ATP Rate Assay Kit was applied. OCR—Oxygen Consumption Rate (shows respiration), ECAR—Extracellular Acidification Rate (shows glycolysis). The same metabolic inhibitors were used as in Figure 10A,B (see description there). Mann–Whitney (*U*-test) was applied to check statistically significant differences * *p* ≤ 0.05; ** *p* ≤ 0.01; ns—non significant.

Figure 10 and Figure 11 demonstrate that, although 20E treatment strongly induced the up-regulation of both glycolysis and respiration, it was not able to overcome the LY294002- mediated inhibitory effect on both metabolic processes in mouse myoblasts and fibroblasts.

Taken together, our data emphasize the importance of the PI3K/AKT/mTOR pathway in the 20E-mediated induction of energy metabolism.

## 4. Discussion

Despite the fact that 20-hydroxyecdysone (20E) is a well-known insect hormone, it has also been shown to possess various pharmacological activities in mammals. These activities include anabolic, adaptogenic, hypoglycemic, antioxidant, cardioprotective, hepatoprotective, and neuroprotective effects on healthy tissues [3]. Furthermore, 20E displays adjuvant antineoplastic manifestation in transformed cells [41,42]. Notably, mammals, unlike insects, lack the G-protein-coupled ecdysone receptor (EcR) which is responsible for 20E-mediated effects. Therefore, the molecular mechanisms underlying 20E-dependent pleiotropic pharmacological activities in mammals are still enigmatic. The only molecular mechanism proposed to date implies the interaction of 20E with both the G-protein-coupled receptor MAS1 and the estrogen receptor (ER), which results in 20E-modulated protein synthesis and myostatin expression in C2C12 myotubes [24]. It should be noted, however, that we observed 20E activity also in triple-negative breast cancer (TNBC) cell lines that lack ER [41], arguing that the wide range of pharmacological activities of 20E in tissues of different origins cannot be determined by only these targets. Moreover, although the anabolic activity of 20E has been widely recognized, the studies were limited to the 20E-mediated up-regulation of protein biosynthesis and the increase in the size of myofibrils, without delving into the molecular events underpinning these phenotypes [4,22,23,24]. Finally, previous studies did not assess the effect of 20E on energy metabolism.

In this study, we sought to uncover the transcriptional and metabolic effects caused by the 20E treatment of C2C12 cells. The latter are a well-characterized model of immortalized mouse myoblasts that help interpretation of the results obtained at the molecular level in the context of muscle function and repair.

The RNA-seq analysis uncovered a large number of genes affected by the 20E treatment of C2C12 myoblasts. Interestingly, c-Myc, the AP-1 transcription factor family members (Jun, Fos, and Atf3) and its downstream effectors, and the transcription factors Egr1, Egr2, and Egr3, were in the top list of genes induced by 20E. c-Myc is known as the master regulator of the metabolic network, affecting different metabolic processes and transactivating almost all genes that code for enzymes of glycolysis and one-carbon metabolism [35,36,37]. ATF3 is considered a ‘’hub of the cellular adaptive-response network’’ [39], regulates serine biosynthesis by increasing PHGDH, PSAT1, and PSPH [38], and also coordinates serine and nucleotide metabolism [40]. The literature data suggest that ATF3 also is an important regulator of glucose and lipid metabolism in various tissues [43]. The metabolic functions of ATF3 in muscle tissues remain largely unknown and need to be explored in further studies. Likewise, the role of AP-1 in skeletal muscle metabolism is still enigmatic; however, it was reported to act together with the Estrogen Related Receptor to activate transcription of the peroxisome proliferator-activated receptor γ coactivator 1α (PGC-1α) [44].

Importantly, we observed a significant number of 20E-upregulated genes involved in protein translation, oxidative phosphorylation, different cellular biosynthetic processes, and stress response. These results are consistent with the literature data on the adaptogenic and anabolic activities of 20E [3].

Increasing the intensity of anabolism requires the coordinated up-regulation of a number of biosynthetic processes, for which glycolysis and one-carbon metabolism are crucial sources of structural units [37,45,46]. Glycolysis provides all non-essential and conditionally essential amino acids [47]. Its intermediate, 3-phosphoglycerate, is a carbon source for serine, glycine, and cysteine, while another intermediate, pyruvate, is a direct source for alanine production. Furthermore, being introduced into the citric cycle, pyruvate paves the way for glutamate, glutamine, asparagine, aspartate, and proline biosynthesis [48].

Glycolysis supplies one-carbon metabolism through serine biosynthesis from 3-phosphoglycerate [37,49]. In turn, one-carbon metabolism functions as a regulator and a sensor of the cells’ nutrient status through the cycling of one-carbon groups and the transferring of them from donors (serine and glycine) to different acceptor compounds, including the intermediate products of the biosynthesis of nucleotides (both pyrimidines and purines), the amino acid cysteine, S-adenosylmethionine (the main donor of methyl group), and glutathione (the main factor of intracellular homeostasis), etc. [50]. As serine is derived from glycolysis intermediate 3-phosphoglycerate, one-carbon metabolism links glycolysis with different biosynthetic processes that result in DNA and RNA biosynthesis, protein biosynthesis, and the maintenance of the redox and methylation states of cells [51,52].

Here, we have demonstrated that 20E increased the expression of a number of enzymes that are involved in glycolysis and one-carbon metabolism. Regarding the latter, 20E induced the expression of two key enzymes, SHMT2 and MTHFD2.

The importance of one-carbon metabolism for muscle cells has been recently illustrated by Mäntyselkä and co-authors [53]. The authors inhibited key enzymes of glycolysis, the pentose phosphate pathway, and the serine synthesis pathway to evaluate their importance in C2C12 myotube protein synthesis. It was the serine biosynthesis pathway that was crucial for skeletal muscles to generate cell biomass, because the inhibition of serine biosynthesis, but not of the other pathways, decreased protein synthesis, myotube size, and the proliferation of myoblast cells in an mTORC1-dependent manner [53].

In line with the molecular data obtained, we have demonstrated for the first time a 20E-mediated increase in both glycolysis and respiration. Generally, the degree of increase in respiration turned out to be greater than the degree of increase in glycolysis, which correlates with the RNA-seq data on the up-regulation of genes involved in oxidative phosphorylation and suggests that increased respiration is not simply a consequence of enhanced glycolysis. In line with this, Baev and co-authors reported that 20E was able to increase mitochondrial coupling and mitochondria membrane potential in isolated rat mitochondria [18]. It is also important to note that 20E augmented both glycolytic and mitochondrial ATP production in our assay. In line with elevated glycolysis, we observed a 20E-dependent increase in glucose uptake, which was also reported by Chen and co-authors in Hep2G hepatoma cell lines [21].

We have also shown that 20E increases global protein biosynthesis in C2C12 myoblasts and NIH3T3 fibroblasts. This is consistent with the previously obtained data on the 20E-mediated increase in protein synthesis in differentiated myotubes [22,24]. It appears that 20E’s anabolic properties extend beyond muscle tissue and could potentially aid in the treatment of various pathologies. Anabolic activity, of course, necessitates a coordinated increase in energy production with improved biosynthetic processes. In fact, we have demonstrated that 20E boosts both catabolism and anabolism, coupling the energy-producing and biosynthetic metabolic processes. This may be the basis for 20E’s well-known anabolic activity.

In light of 20E’s impact on anabolic processes, other biosynthetic pathways remain to be studied. In this respect, HMP shunt (aka PPP) is especially important, as it is responsible for the production of the ribose and NADPH that are required for nucleotide biosynthesis, de novo lipogenesis, and redox homeostasis.

It is important to note that the 20E-induced activation of AKT kinase occurs in various tissues of both insects and mammals [17,20,22]. Moreover, using a pan-inhibitor of G-protein-mediated signaling, Gorelick and co-authors demonstrated the involvement of G-proteins and AKT in the 20E-dependent effects in C2C12 cells [22]. Taken together, these data point to a conservative mechanism of 20E action that involves G-proteins and AKT activation.

In accordance with this finding, the present study demonstrates that 20E induces the activation of both AKT (S473) and mTOR (S2448). Moreover, the use of the PI3K inhibitor LY294002 clearly revealed the importance of the PI3K/AKT/mTOR pathway in the 20E-mediated induction of glycolysis and respiration. In turn, a number of studies have demonstrated the important roles of both AKT and mTOR in up-regulating glycolysis and respiration in various tissues [54,55,56,57,58].

In contrast to myoblasts and fibroblasts, we have previously revealed the 20E-dependent inhibition of glycolysis and respiration in breast carcinoma and non-small cell lung cancer cell lines [41,42]. Although the underlying molecular mechanisms remain elusive, it seems that the beneficial pharmacological activities of 20E can also be complemented by antitumor ones, as metabolic rewiring is considered to be one of the “hallmarks of cancer” [10,59,60].

In terms of clinical translation, 20E seems to be a very suitable adjuvant for anticancer therapy, as it is a low-toxic compound with an LD50 > 9 g/kg in mice [3]. Recently, Dioh and co-authors [61], investigated the safety and pharmacokinetics of 20E in healthy young and older adults in both the single ascending dose and the 14-day multiple ascending dose modes. These authors demonstrated that a single ingestion of 700–1400 mg of 20E led to a plasma concentration of 350–650 ng/mL, which approximately corresponds to 0.73–1.35 μM. The latter is within the range of doses that showed physiological effects on C2C12 cells in our studies. Moreover, 20E is stable in water solution [62] and can be detected in post-administration urine samples for more than 2 days [63]. Due to these reasons, 20E may be considered as a potent adjuvant that mitigates the adverse effects of common anticancer therapies.

Furthermore, the results of the present study strongly argue that 20E properties should be investigated not only in tumor cells but also in normal cells because of its adaptogenic, anabolic, antioxidant, hepato-, cardio-, and neuroprotective activities in healthy non-cancer tissues [3,5,7,9] due to its anabolic, adaptogenic, antioxidant, and other valuable pharmacological activities.

## 5. Conclusions

In the present study, we observed the 20E-dependent up-regulation of both catabolism and anabolism, coupling energy-producing and biosynthetic metabolic processes in mouse myoblasts and fibroblasts.

## Data Availability

RNA-seq raw data are deposited in the Mendeley data repository divided in two parts: https://data.mendeley.com/datasets/66x9dj3w7v/1 (https://doi.org/10.17632/66x9dj3w7v.1; accessed on 1 March 2024) and https://data.mendeley.com/datasets/tbynrzn5sd/1 (https://doi.org/10.17632/tbynrzn5sd.1; accessed on 1 March 2024).

## Supplementary Materials

The following supporting information can be downloaded at: https://www.mdpi.com/article/10.3390/antiox13111349/s1, Figure S1. KEGG PATHWAY Database profiling (https://www.genome.jp/kegg/pathway.html, accessed on 25 February 2024) for genes up-regulated by 20E in C2C12 myoblasts revealed approximately 30 genes coding for components of all mitochondrial respiratory complexes. Figure S2. Top 30 genes down-regulated by the treatment of C2C12 myoblasts with 1 μM of 20E for 24 h (RNA-seq data). Control–control samples (DMSO-treated); 20E-samples treated with 20E. Figure S3. The analysis of the protein–protein interaction network for up-regulated genes using the STRING database (https://string-db.org; accessed on 25 February 2024). Colors indicate protein participation in the corresponding cellular process. Figure S4. 20E increased both glycolysis and respiration in C2C12 myoblasts and NIH/3T3 fibroblasts. The SeaHorse XF Real-Time ATP Rate Assay Kit was applied. SeaHorse OCR and ECAR plots are presented. OCR—Oxygen Consumption Rate (shows respiration); ECAR—Extracellular Acidification Rate (shows glycolysis). Mann–Whitney (U-test) was applied to check statistically significant differences * *p* ≤ 0.05; ** *p* ≤ 0.01. Table S2. The list of differentially expressed genes upon treatment of C2C12 myoblasts with 1 μM of 20E for 24 h.
