# Supplementary material for: 20-Hydroxyecdysone Boosts Energy Production and Biosynthetic Processes in Non-Transformed Mouse Cells
Source: Antioxidants (Basel). 2024 Nov 2;13(11):1349. doi: 10.3390/antiox13111349 (PMC11591052; doi:10.3390/antiox13111349)
Supplement: Supplementary file 1 [file antioxidants-13-01349-s001.zip › Table S1.pdf]

The list of primers used for Real-Time PCR

Gene	Forward primer	Reverse primer
**LDHA** (NM_010699.2)	CAGACTTGGCTGAGAGCATAA	GATACATGGGACACTGAGGAAG
HK2 (NM_013820.4)	GCTGGAGGTTAAGAGAAGGATG	TGGAGTGGCACACACATAAG
**SLC2A1** (Glut1)(NM_011400.3)	TATCAGCCACTCTCCTATCTCC	AGGTCCAGCCCTACAGATTA
**ALDOA** (NM_001177307.1)	ACCAATGGCGAGACAACTAC	GTCGGCTCCATCCTTCTTATAC
c-Myc (BC138931.1)	CTGGAGATGATGACCGAGTTAC	GAGAAACCGCTCCACATACA
**PSAT1** (NM_177420.2)	GAGAAGCTCAGCTCCATCAAA	GCTCCACTGGGCATACATAAA
**PSPH** (NM_133900.4)	GCAGTGTGCTTTGATGTTGATAG	ATGGCTCTCCGTGTCATTTC
**PHGDH** (NM_016966.3)	GAGGAGGCATTGTGGATGAA	CGTGGTGGCTCTTCTGTAAA
**SHMT2** (NM_028230.4)	GACCCGGAAGTTACCTTTCTT	CCTGGCTCTTGCCCTAAAT
**MTHFD2** (NM_008638.2)	GGGTGCTTTGAGGCCTATTT	AGCTGCTGACAGTTCTTTGG
**ATF3** (NM_007498.3)	CTCCTGGGTCACTGGTATTTG	CCGATGGCAGAGGTGTTTAT
**β-actin** (NM_007393.5)	CAGCCTTCCTTCTTGGGTATG	GGCATAGAGGTCTTTACGGATG
