# Supplementary figures and images for: 20-Hydroxyecdysone Boosts Energy Production and Biosynthetic Processes in Non-Transformed Mouse Cells
Source: Antioxidants (Basel). 2024 Nov 2;13(11):1349. doi: 10.3390/antiox13111349 (PMC11591052; doi:10.3390/antiox13111349)

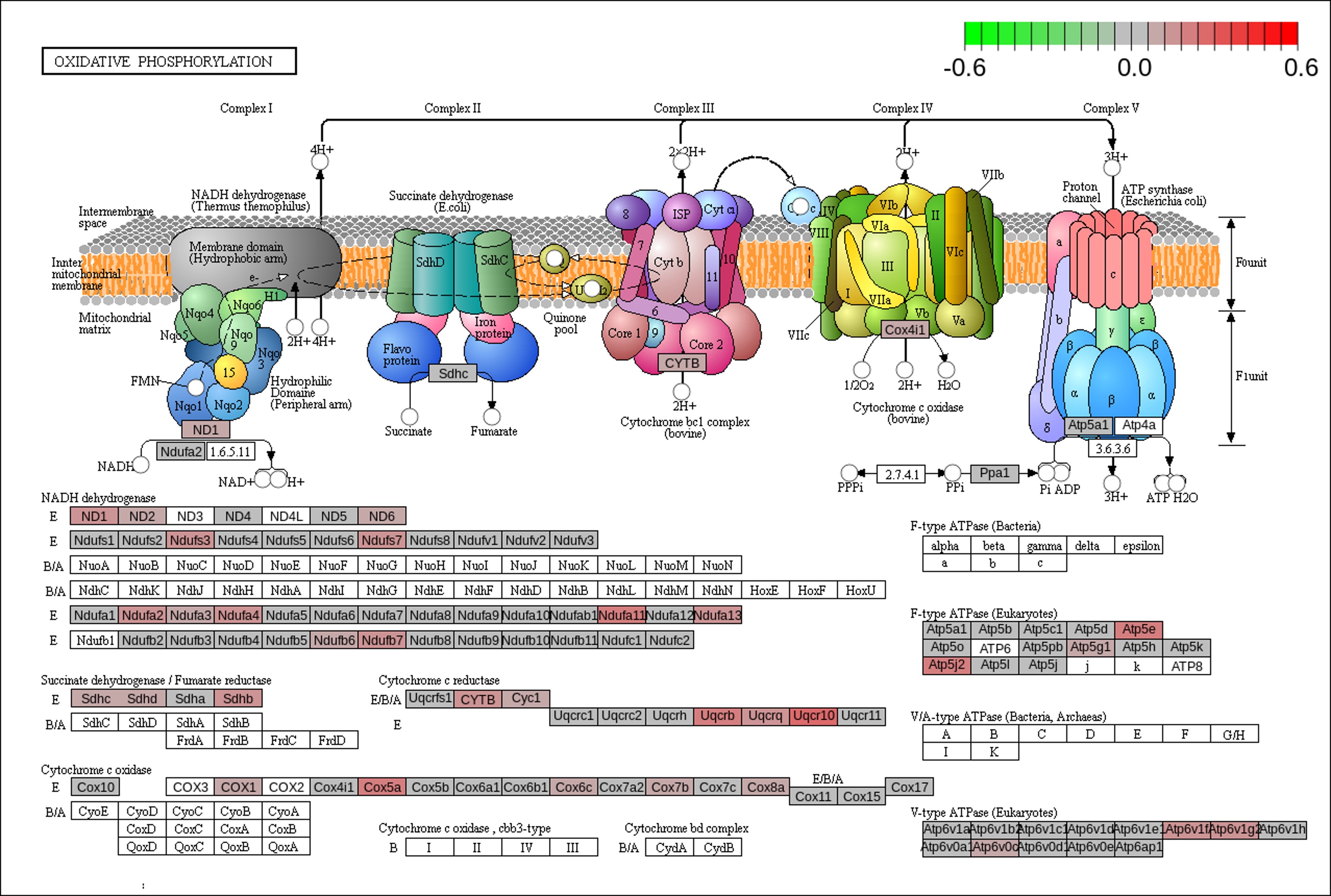

Supplement: Supplementary file 1 [file antioxidants-13-01349-s001.zip › Figure S1.jpg]

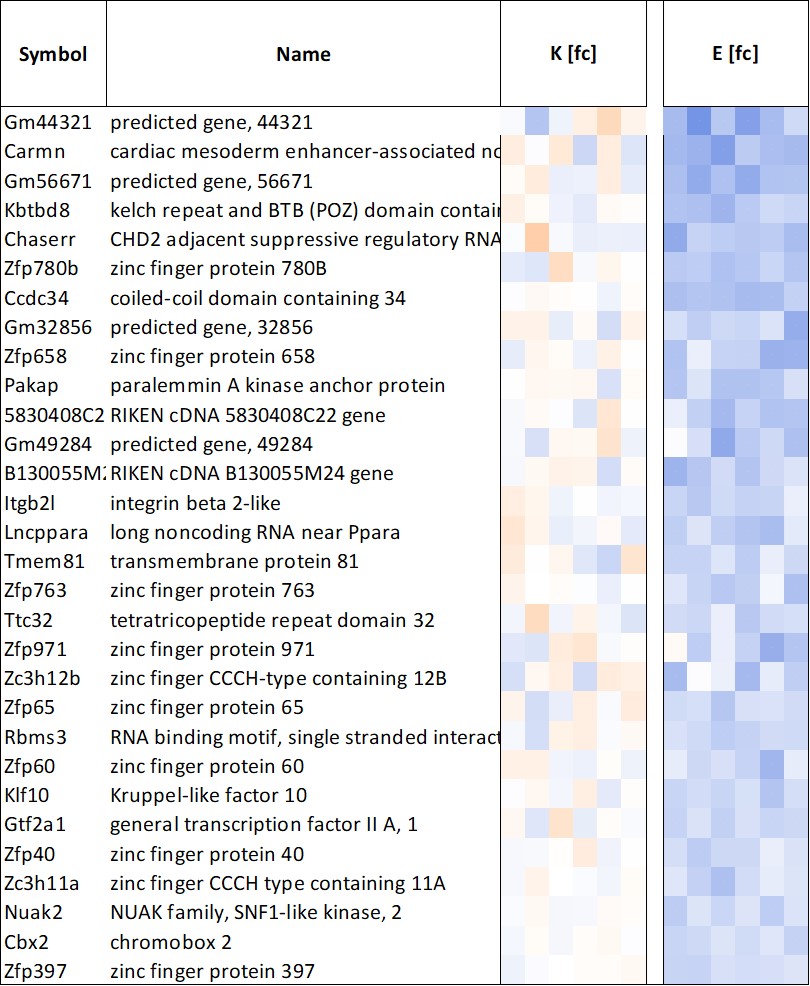

Supplement: Supplementary file 1 [file antioxidants-13-01349-s001.zip › Figure S2.jpg]

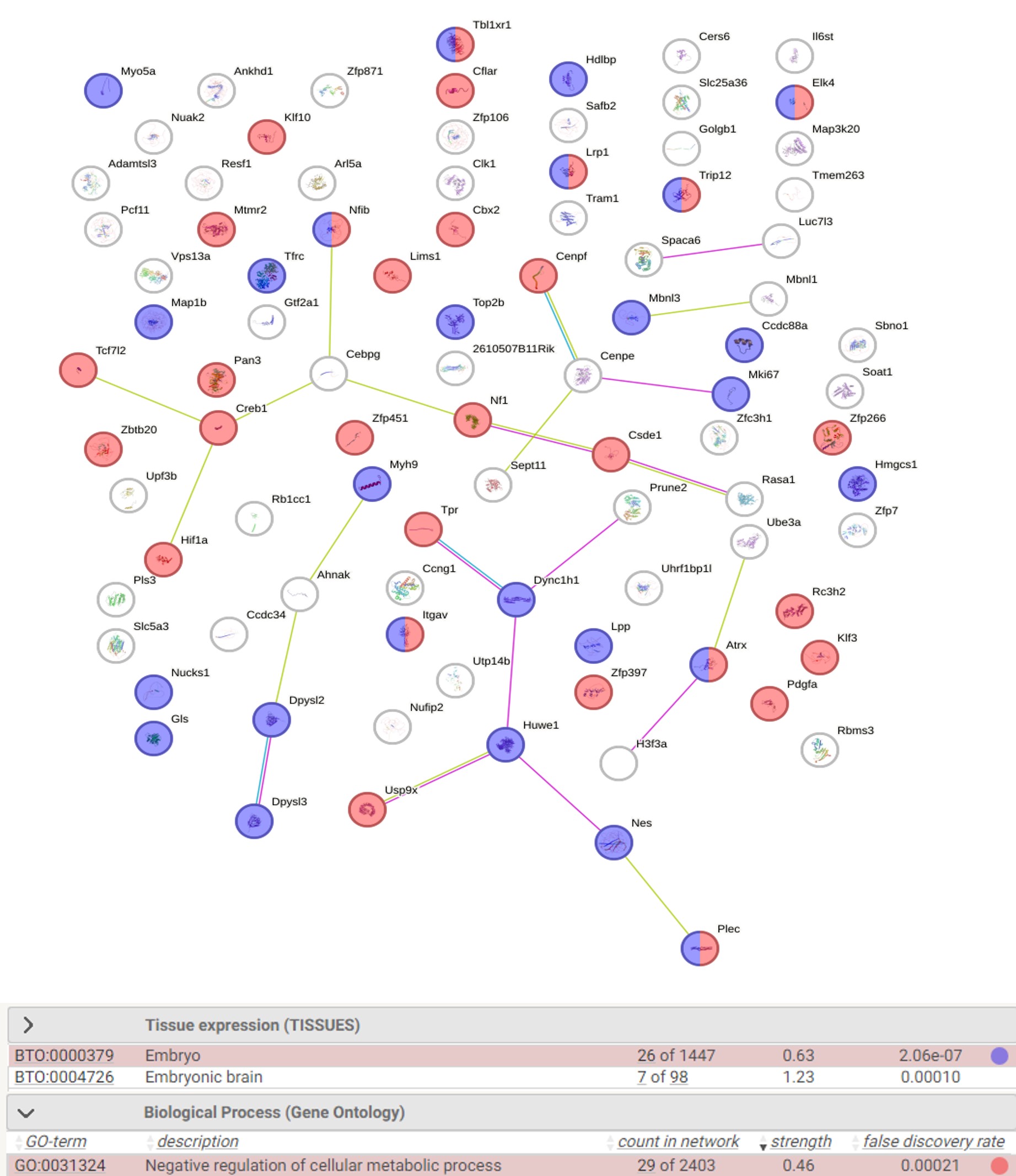

Supplement: Supplementary file 1 [file antioxidants-13-01349-s001.zip › Figure S3.jpg]

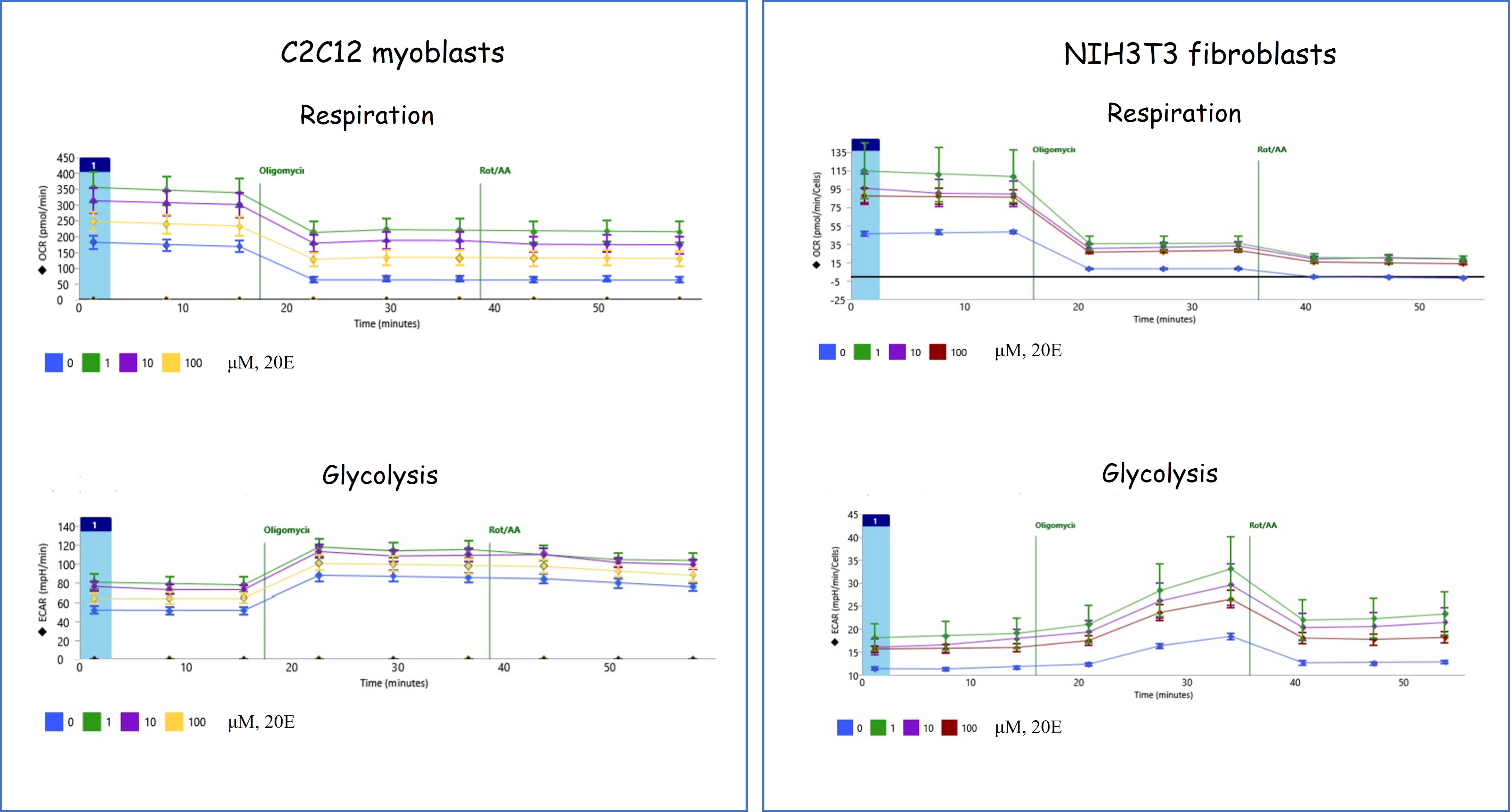

Supplement: Supplementary file 1 [file antioxidants-13-01349-s001.zip › Figure S4.jpg]
